# *In vivo* Neural Crest Cell Migration Is Controlled by “Mixotaxis”

**DOI:** 10.3389/fphys.2020.586432

**Published:** 2020-11-25

**Authors:** Elias H. Barriga, Eric Theveneau

**Affiliations:** ^1^Mechanisms of Morphogenesis Lab, Instituto Gulbenkian de Ciência, Oeiras, Portugal; ^2^Centre de Biologie du Développement (CBD), Centre de Biologie Intégrative (CBI), Université de Toulouse, CNRS, UPS, Toulouse, France

**Keywords:** directed cell migration, neural crest, morphogenesis, durotaxis, chemotaxis, galvanotaxis, electrotaxis, mixotaxis

## Abstract

Directed cell migration is essential all along an individual’s life, from embryogenesis to tissue repair and cancer metastasis. Thus, due to its biomedical relevance, directed cell migration is currently under intense research. Directed cell migration has been shown to be driven by an assortment of external biasing cues, ranging from gradients of soluble (chemotaxis) to bound (haptotaxis) molecules. In addition to molecular gradients, gradients of mechanical properties (duro/mechanotaxis), electric fields (electro/galvanotaxis) as well as iterative biases in the environment topology (ratchetaxis) have been shown to be able to direct cell migration. Since cells migrating *in vivo* are exposed to a challenging environment composed of a convolution of biochemical, biophysical, and topological cues, it is highly unlikely that cell migration would be guided by an individual type of “taxis.” This is especially true since numerous molecular players involved in the cellular response to these biasing cues are often recycled, serving as sensor or transducer of both biochemical and biophysical signals. In this review, we confront literature on *Xenopus* cephalic neural crest cells with that of other cell types to discuss the relevance of the current categorization of cell guidance strategies. Furthermore, we emphasize that while studying individual biasing signals is informative, the hard truth is that cells migrate by performing a sort of “mixotaxis,” where they integrate and coordinate multiple inputs through shared molecular effectors to ensure robustness of directed cell motion.

## Introduction

Finding a solution to trigger directed cell migration is simple. An external signal that cells can interpret needs to be spatially organized. Then, cells can use that signal to generate a front–rear polarity allowing directional movement along that cue. Very much like drivers following road signs. Many inputs (e.g., chemical, mechanical, electrical, topological) can be shown to fulfill this function in controlled and simplified experiments (Zhao et al., 2006; Capuana et al., 2020; Zhu et al., 2020). However, living systems were not engineered by a designer to strictly follow a set of specifications in a logical manner that is then validated by external quality controls. Instead, *in vivo* migrating cells are often exposed to an overwhelming range of inputs which may at best appear to have no obvious hierarchy and at worst to be contradictory. Yet, the migratory response of cells to such convoluted environments is still logical. In addition, each polarity cue may not be as neatly organized as it would in an *in vitro* assay. Further, some cells may display a given migratory behavior while their neighboring tissues do not. Hence, there may be cooperation, coordination and/or competition between directionally migrating cells and the activities of their neighbors. Furthermore, a given input may lead to different responses in different cell populations within the same time window indicating that the directional information is not carried by the signal itself but generated as a result of the interplay between cells and a given signal or set of signals (we discuss examples hereafter). This can be equated to how geneticists view the phenotype as a result of the interaction between a genotype and the local environment of an organism. Yet, for cells willing to undertake directed migration, it all comes down to two simple facts: (i) cells need to propel themselves and (ii) establish and sustain a front–rear polarity. This means that all inputs have to be somewhat integrated by a cell for a directional behavior to emerge. In groups of cells, intercellular communication may in addition lead to emerging properties such that what a cell collective does may differ from what a single cell would do in a similar context (Theveneau et al., 2010). Hence, unveiling the mechanisms that control directed cell migration in its full complexity could have countless impacts in our understanding of intricate morphogenetic events. In addition, a more integrative approach to directed cell migration would help designing effective ways to hinder cancer metastasis, improve wound healing or contribute to new methods for *ex vivo* organ patterning in the context of regenerative medicine. In this review, we used the Xenopus cephalic neural crest (NC) cells, an embryonic stem cell population that collectively and directionally migrates (Gouignard et al., 2018), as an example to discuss the complexity of the control of directed cell migration. We address first how motility is initiated in NC cells before discussing the strategies displayed by cells in order to bias their motion and perform directed cell migration. Drawing parallels between NC results and findings about directed cell migration in other cell types, we propose some working hypotheses for signal integration and the emergence of directional motion.

## The Neural Crest, EMT, and the Onset of Cell Motion

**FIGURE 1:**
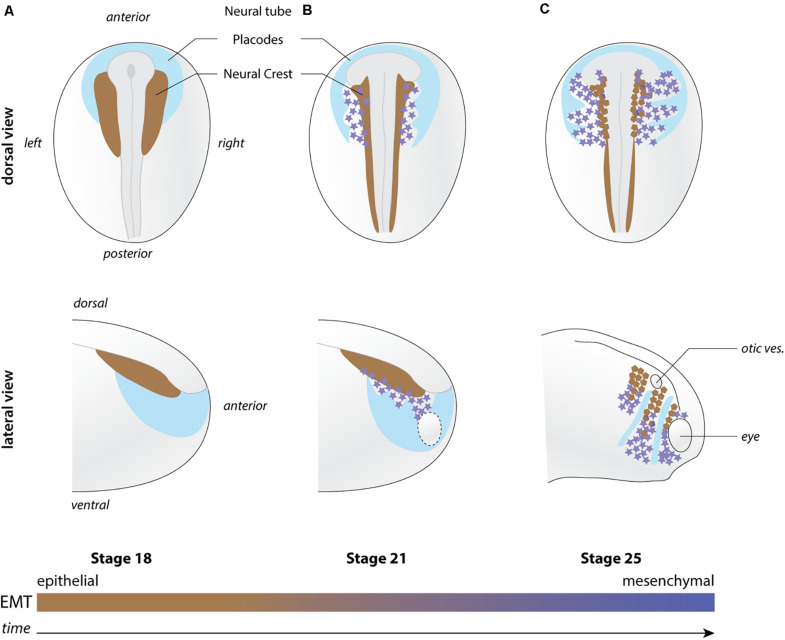
Overview of neural crest migration. **(A–C)** Diagrams depicting the position of NC cells (shades of brown to magenta) with respect to the placodal region (light blue) at pre-migration (stage 18), early migration (stage 21), and late migration (stage 25). EMT is progressively implemented as NC migration proceeds. Brown NC cells are more epithelial while magenta star-shaped NC cells are more mesenchymal. Top and bottom rows shows lateral views and dorsal views, respectively. Orientations and structures are indicated on the figure. Ot. ves., otic vesicle.

NC are induced during mid to late gastrulation stages at the interface between the neural and non-neural ectoderm and between the epidermis and mesoderm (Figure 1). They later leave the dorsal neuroepithelium to collectively migrate throughout the developing embryo. Anterior NC cells make an outstanding contribution to the head morphology and sensory structures by providing cartilage and bones, meninges that surround the brain, smooth, and striated muscle cells and tendons as well as pigments cells among other structures (Dupin et al., 2006). In addition, NC cells cooperate with placodal cells to form the cephalic peripheral nervous system (Theveneau and Mayor, 2011). Cranial placodes are discrete thickenings of the ectoderm that produce some of the neurons that in turn form the cranial ganglia (Schlosser, 2014). The rest of the neurons and the glial cells are provided by the cephalic NC cells (Theveneau and Mayor, 2011). NC cells are an extremely powerful model to investigate cell migration. Their timing and pattern of migration has been documented in multiple species allowing comparative studies (Theveneau and Mayor, 2012). In chicken, mice and Xenopus embryos, NC cells can be manipulated *in vivo* and *ex vivo*, thanks to well-defined culture conditions. This has allowed researchers to perform in-depth cell and molecular biology studies. Whereas in genetically tractable species (e.g., zebrafish and mouse), transgenic lines have been generated for long-term observation and targeted molecular manipulation of these cells. In addition, the first part of NC cell migration occurs superficially, especially in cephalic regions, permitting direct observation of cell behavior by time-lapse cinematography in fish, chick or amphibians.

The first step toward directed motion is for cells to acquire motile capabilities. NC cells initiate migration by undergoing epithelial-mesenchymal transition (EMT). EMT leads to a qualitative and quantitative remodeling of adhesive properties, cytoskeleton dynamics and cell polarity such that cells have transient adhesions to one another, display faster membrane dynamics and go from apicobasal polarity associated with epithelial state to a front–rear polarity associated with motility. EMT is performed by a series of non-obligatory steps such that cells that initiate EMT do not systematically complete it (Yang et al., 2020) and is reversible (Pei et al., 2019). EMT in Xenopus NC cells is better described as a partial EMT with cells migrating at high cell density with frequent transient physical contacts via functional adherens junctions, as recently discussed (Gouignard et al., 2018).

Canonical EMT is controlled by an array of transcription factors whose expression is detected many hours before NC migration is initiated. In *Xenopus*, cephalic NC migration starts around stage 19–20 when the neural folds closure nears completion to form the neural tube. Nonetheless, the expression of key EMT transcriptional regulators such as Snail2 or Twist1 starts in NC cells at stages 12 and 14, respectively. One of the main targets of these factors is the cell–cell adhesion receptor E-cadherin (CDH-1) whose expression only starts to decrease in the NC at around stage 17.5 (Scarpa et al., 2015), suggesting that Snail2 and Twist1 may not be recruited to the E-cadherin promoter or that they may not even be active until stage 17.5. One way to control transcription factors’ activity is to regulate their entry into the nucleus. Intriguingly, in mammalian cell lines, Twist has been shown to be imported to the nucleus when cells are exposed to stiff substrates (Wei et al., 2015; Fattet et al., 2020). In this situation, EphA2 is activated in a ligand-independent manner and leads to the phosphorylation of Twist via LYN kinase. This frees Twist from its cytoplasmic anchor G3BP2 and allows it to enter the nucleus. This is particularly interesting in the context of NC development because the onset of NC migration in Xenopus has been linked to the local increase of stiffness underneath the cephalic crest generated by the convergent extension movement of the mesoderm toward the midline of the embryo (Barriga et al., 2018). In addition, Twist expression is under the control of the Hif signaling pathway which also controls the expression of CXCR4, the receptor for the chemokine CXCL12/Stromal cell-derived factor 1 (Sdf1) (Barriga et al., 2013). Interestingly, in renal carcinoma cells, Hif1α and CXCR4 have been shown to take part in a feed forward loop for nuclear translocation such that, via a direct physical interaction between the two proteins, nuclear accumulation of CXCR4 favors entry of HIF-1α and HIF-1α then further promotes CXCR4 expression (Bao et al., 2019). Thus, one can propose a model in which Hif-1α primes NC cells for EMT and directional migration by regulating Twist and CXCR4 expressions until mesoderm stiffness reaches a threshold suitable for migration. Twist1 is not the main and certainly neither the only EMT-associated NC transcription factor, however, to date it is the most likely candidate to mediate a “rapid” response to environmental cues. Another example is that of Sox10, this transcription factor constantly shuttles between the nucleus and the cytoplasm and docks at the surface of mitochondria (Rehberg et al., 2002; Mou et al., 2009). However, experimental assessment of Sox10’s function ties it to lineage decisions rather than NC migration in Xenopus (Aoki et al., 2003; Honore et al., 2003). Whether Sox10’s nuclear localization is also mechanically controlled remains to be explored. In any case, controlling the emergence of cell motility does not explain directionality *per se*. Such cell intrinsic motility needs to be iteratively biased to sustain directed motion. The rest of this review is dedicated to the various cues that might bias NC directed motion.

## Chemotaxis

**FIGURE 2:**
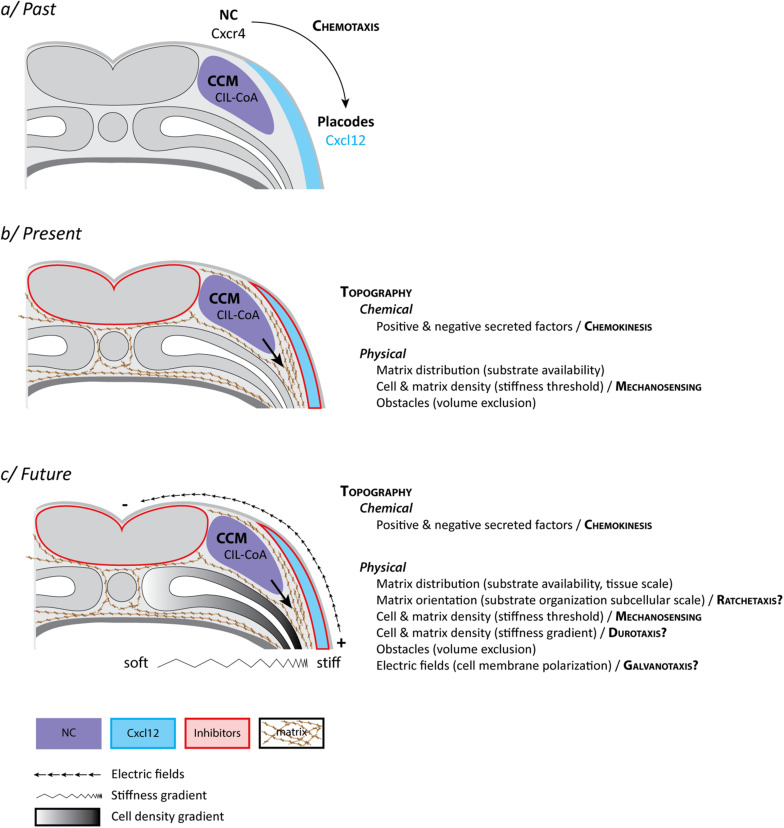
Neural crest “mixotaxis.” **(a)** The classical view of cephalic NC cell directed cell migration in *Xenopus laevis*. NC cells become motile via EMT and exhibit a collective behavior [collective cell migration (CCM)] due to a balance between dispersion (CIL) and mutual attraction (or co-attraction, CoA). Placodes, located in the lateral ectoderm, produce CXCL12, a well-known chemoattractant. NC cells express the main CXCL12 receptor, Cxcr4. NC are migrating toward latero-ventral territories due to CXCL12-dependent chemotaxis. **(b)** The current view of cephalic NC cell directed cell migration in *Xenopus laevis* in which CXCL12, by promoting cell-matrix adhesion, contributes to defining permissive areas for cell migration in the context of a biased distribution of topological features. These include chemical and physical cues and requires a minimal stiffness of the surrounding tissue for cell migration to proceed. The main difference with the classical view is that precise and biased spatial distribution of secreted molecules is dispensable. **(c)** A speculative view of what the actual control of cephalic NC cell directed cell migration in *Xenopus laevis* might look like with the inclusion of additional features such as a hypothetical graded distribution of stiffnesses (Durotaxis) and electric fields (Galvanotaxis) at tissue scale as well as iterative biases in topography at cellular and subcellular scales (Ratchetaxis). While most of these features can be experimentally disentangled under controlled *ex vivo* experiments, none of these cues relies on a specific set of molecular sensors and effectors but rather share downstream signal transduction machineries leading to cell adhesion and polarity. Therefore, *in vivo*, each input (e.g., chemical, mechanical, electrical) is likely to extensively feed into the others leading to the exciting idea that, in their native environment, NC cells may achieve directed migration by performing a sort of “mixotaxis.” See main text for details.

The directional migration of NC cells could be explained by chemotaxis, the ability of cells to follow gradients of soluble guidance cues (Figure 2a). As mentioned, NC and placodes cooperate to form the cephalic peripheral nervous system. Interestingly, NC and placodes interact early on during head morphogenesis and this interaction is crucial for directional migration of NC cells (Culbertson et al., 2011; Theveneau et al., 2013). Prior to the onset of NC migration, NC and placodes are located in adjacent domains of the lateral ectoderm. NC are on either side of the neural plate and the placodes are surrounding the NC domains and the anterior neural plate, forming a horseshoe-shaped zone (Figure 1). Placodes secrete CXCL12 that promotes cell-matrix adhesion and motility via activation of Rac1 in NC cells (Theveneau et al., 2010, 2013; Bajanca et al., 2019). The presence of this chemokine stimulates migration such that NC move toward the CXCL12-producing placodes. When NC cells and placodes make a physical contact they exhibit contact-inhibition of locomotion (CIL), an active repolarization process upon cell–cell contact that leads to cells moving away from each other (Stramer and Mayor, 2017). However, placodes and NC cells do not have the same migratory capabilities. Placodes at this stage are epithelial, located in the deep layer of the ectoderm and are barely motile. NC cells being more active, they are systematically the ones filling local gaps between cells generated by the CIL response. This creates a bias that favors lateral migration of the crest cells toward the placode domain. Thus, once NC migration is initiated there is a progressive shift of the placodal cells laterally/ventrally that displaces the source of CXCL12. This has been proposed to generate a feed forward loop driving directed movement of both cell populations from medial to lateral (Theveneau et al., 2013; Figure 1). At first glance, this mechanism explains the directional movement of the NC cells and the progressive redistribution of placodes during head morphogenesis via a combination of CXCL12-dependent chemotaxis and heterotypic contact-inhibition between NC and placodes. So, what is missing?

There are several caveats. First, we infer lots of *in vivo* directional migratory behaviors and mechanisms (chemotaxis, haptotaxis, ratchetaxis, durotaxis) from *in vitro* data which in general show that cells have the ability to interpret and follow such signals. Nonetheless, clear demonstration of their actual implication in directed cell migration *in vivo* is tough, owing to the complex nature of native environments. Some of these directional cues are also not easy to distinguish from one another. In particular, it is difficult to assess whether cells undergo chemotaxis (soluble signal) vs. haptotaxis (bound signal) *in vivo*. For instance, CXCL12 and VEGFA, common examples of putative NC chemotactic cues (McLennan et al., 2010; Theveneau et al., 2010), are capable of binding to the extracellular matrix and we still do not understand whether their physiological relevance is linked to a soluble or a bound state. Also, graded distribution of a signal is not a proof that cells are detecting it or reading it. In the case of CXCL12 and VEGFA such unequivocal proof of graded distribution of the protein along migratory paths has not been obtained. Moreover, while CXCL12 is a powerful chemotactic factor for NC *in vitro* (Theveneau et al., 2010), its spatial distribution is dispensable *in vivo* as it primarily acts by promoting adhesion to the extracellular matrix rather than giving clear direction to the cells (Bajanca et al., 2019). This has been demonstrated by showing that *in vivo* directed NC migration can occur in the absence of CXCL12/CXCR4 signaling if Rac1 is homogenously and iteratively activated in NC cells to allow for cell-matrix adhesions to form (Bajanca et al., 2019). This suggests that CXCR4-CXCL12 may work as a chemokinetic factor (promoting motility via cell-matrix adhesion) rather than a chemotactic one (biasing directionality). If CXCR4-CXCL12 signaling does not provide a directional bias what are the mechanisms ensuring sustained directed motion and how does CXCR4-CXCL12 integrate with them?

## Durotaxis

Durotaxis is the directed motion of cells according to local gradients of rigidity (stiffness) with cells moving from compliant to rather stiff regions of a given substrate (Lo et al., 2000). For example, in Xenopus, cell proliferation drives local changes in brain tissue stiffness, creating local gradient that are followed by axons of developing neurons (Thompson et al., 2019). Given that NC cells are able to sense differences in rigidity and that stiffness of the underlying mesoderm is a key factor for the initiation of NC migration (Barriga et al., 2018), one could also propose that there might be a gradient from dorsal to ventral promoting stiffness-dependent directional migration. The main driver of this observed increase of stiffness is the local accumulation of mesodermal cells underneath the NC domain (Barriga et al., 2018). In the trunk, the medio-dorsal mesoderm aggregates as somites and thus is denser than the ventro-lateral mesoderm (Figure 2). Therefore, if there is a cell density associated gradient of mesoderm stiffness it would be oriented ventro-dorsally which is opposite to the direction of trunk NC migration. In the head, where mesoderm does not form somites, such spatial distribution of cell density and stiffness has not been assessed so far. Though, published data suggest that the emergence of such a gradient is unlikely owing to the high degree of mechanical heterogeneities observed in that region (Barriga et al., 2018). Yet, even if true, such gradient of stiffness leading to durotaxis could not be seen as an absolute signal that would restrict any kind of cell movement in a dorsoventral manner. While cephalic NC cells are migrating ventralward, the surface ectoderm is moving dorsalward to accompany dorsal neural tube closure. In addition, myeloid cells (macrophages) are undergoing random migration from the cardiac region to survey the entire developing embryo (Agricola et al., 2016). Myeloid cells migrate as single cells, display extensive dispersion, and cross areas that NC cells are completely unable to use. Thus, during head morphogenesis, there are concomitant migration events (e.g., epidermis, neural crest, myeloid cells) that follow different directions despite sharing a common environment. This highlights the importance of considering the interaction between cells and the environment as the main driver of cell behavior rather than intrinsic cell motility.

Mechanosensing of the substrate requires functional cell-matrix adhesions. Thus, in NC cells, the putative distinction between durotaxis and chemotaxis/chemokinesis downstream of CXCR4 is further blurred by the fact that CXCL12 regulates cell-matrix adhesion (Bajanca et al., 2019). This does not mean that CXCR4 is involved in mechanosensing in NC cells. Instead, we could see CXCR4 signaling as priming cells to undergo mechanosensing by allowing them to functionally interact with the matrix. Interestingly, cell-matrix adhesion in cephalic NC cells also involves cadherins (Huang et al., 2016; Langhe et al., 2016). There is an indirect role such that contact-dependent cell polarity primes NC cells to respond to CXCR4 signaling (Theveneau et al., 2010). But there is also a direct role of cadherins. During migration, inhibiting E-cadherin affects adhesion to fibronectin rather than cell–cell adhesion (Huang et al., 2016) and cadherin-11 actively contributes to the formation of focal adhesion (Langhe et al., 2016). This means that we should regard EMT as a way to coordinate the quantitative and qualitative changes in cell–cell and cell-matrix adhesions rather than as a mechanism for cell dispersion in which loss of cell–cell adhesion and motility would be regulated in parallel and as purely cell autonomous properties.

Cadherins take part in regulating cell-matrix adhesions (directly and indirectly) and cell-matrix adhesions are needed to sense substrate stiffness. In turns, when substrate stiffness reaches a threshold it promotes Twist nuclear entry which favors cadherin repression. One wonders about the molecular control of such intricate feedback loops. It could also mean that what has been labeled as CXCR4-dependent chemotaxis might be part of a global change of adhesive property taking place during EMT that prepares cells for efficient stiffness sensing. The existence of stiffness gradients around the cephalic NC cells is still highly speculative. But do cells need such spatially organized mechanical cue to promote directed movement? If so, how could we distinguish durotaxis from the so-called chemotaxis?

## Confinement, Topological Biases, and Ratchetaxis

During EMT, cells pass from stable to transient cell–cell adhesions and this favors cell dispersion *in vitro*. This is further accentuated by CIL that biases cell’s front–rear polarity such that cells move away from cell–cell contacts. However, *in vivo*, cephalic NC cells migrate at high cell density and do not undertake widespread dispersion despite EMT and CIL. The reason for this is that NC cells actively sense and follow each other via complement factor C3a signaling (Carmona-Fontaine et al., 2011) and are constrained by their surrounding tissues physically and chemically (Szabo et al., 2016). When NC cells initiate migration, there are several epithelial structures around them such as the neural plate/tube, the eye, the epidermis, and the cranial placodes. Placodes, as discussed above, are slowly displaced by NC cells such that they organize as discrete structures forming dorsoventral corridors restricting NC migration (Figure 1). This is reinforced by the fact that placodes are also the source of negative regulators of NC motility such as semaphorins rendering their vicinity non-permissive for migration (Yu and Moens, 2005; Bajanca et al., 2019). Interestingly, physical and chemical confinement together with intrinsic motility, CIL and mutual attraction are sufficient to drive directed NC migration even in absence of a stiffness gradient or a chemotactic cue (Szabo et al., 2016; Szabó et al., 2019).

Another putative level of signaling interplay in this context is related to the fact that CXCR4 can physically interact with C3aR, the receptor of C3a, the chemokine mediating NC cell gregarious behavior (Carmona-Fontaine et al., 2011). C3 signaling can enhance CXCR4 signaling and both receptors colocalize in lipid rafts (Honczarenko et al., 2005; Ratajczak et al., 2006; Wysoczynski et al., 2007). Interestingly, lipid rafts are mechanosensitive (Fuentes and Butler, 2012). Thus, stiffness of the mesoderm underlying cephalic NC cells may also modulate a putative C3aR/CXCR4 cooperative signaling by promoting lipid raft remodeling. C3a-dependent mutual attraction increases the likelihood of transient cell–cell contacts. These contacts are known to block Rac1 activity at the site of transient junctions but also to promote an overall increase of Rac1 level in the cells (Theveneau et al., 2010; Carmona-Fontaine et al., 2011). In addition, CXCR4 also promotes Rac1 activity and Rac1 is a key factor in protrusion and focal adhesion formation in cephalic NC cells (Theveneau et al., 2010). Thus, a lack of mutual attraction might also reduce the ability of NC cells to sense substrate stiffness (by lowering the ability to polarize and form cell-matrix adhesions) and might act a selection mechanism to prevent extensive migration of cells that are unable to properly interact with one another. A similar hypothesis could be drawn from the fact that N-cadherin-deficient cephalic NC cells disperse better *in vitro* but fail to polarize efficiently, do not migrate extensively *in vivo* and show signs of weaker cell-matrix adhesion (Kuriyama et al., 2014). This is even more relevant knowing that, in other cells, N-cadherin junctions can be regulated by the association of N-cadherin with lipid rafts and F-actin (Causeret et al., 2005). Thus, cross-regulating cell–cell interaction (N-cadherin and C3) and cell-matrix adhesion (Rac1, CXCR4) in a stiffness-dependent manner during collective cell migration may be a robust way to ensure that only functional cells can efficiently travel together to their final location.

Another level of integration could be mediated by proteases. Xenopus NC cells express Matrix Metalloproteinase MMP14 (a.k.a. MT1-MMP) (Tomlinson et al., 2009; Garmon et al., 2018). Interestingly, MMP14 can cleave Fibronectin (Shi and Sottile, 2011) the main substrate of cephalic NC migration but also inactivates CXCL12 by removing a few of its N-terminal aminoacid (McQuibban et al., 2001). This is even more interesting knowing that CXCL12 exhibit a high binding affinity for Fibronectin (Pelletier et al., 2000). Therefore, Xenopus cephalic NC cells could use MMP14 to remodel Fibronectin (e.g., organization, density), release CXCL12 from the matrix (haptotaxis vs. chemotaxis/chemokinesis) and inactivate CXCL12. This would further crosslink CXCR4-dependent cell-matrix adhesion with mechanosensing and blurs the lines between chemo and haptotaxis.

Xenopus cephalic NC cells are clearly exposed to a topologically biased environment at the onset of migration favoring ventralward migration. The medial part of the embryo with the neural plate/tube acting as an epithelial obstacle which releases several inhibitors of migration and a lower content in fibronectin than the lateral regions (Bajanca et al., 2019) is definitively an unfavorable territory for migration. However, it is unclear if *in vivo* cells experience repeated geometrical or mechanical anisotropy in environment organization known to generate ratchetaxis (Caballero et al., 2015). A more relaxed view of this concept relies on repeated topological anomalies (e.g., repetition of narrow and large spaces) that cells have to cross (Reversat et al., 2020). An important difference between topological bias and confinement as discussed above and ratchetaxis or its declinations is the scale at which these mechanisms act. The aforementioned chemical/physical topological bias acts at tissue scale, defining broad domains that are unsuitable for migration, whereas ratchetaxis occurs as the single cell level or subcellular level biasing individual cell polarity and cytoskeleton dynamics. We currently do not have tools to investigate whether ratchetaxis and the likes are indeed physiologically relevant for Xenopus NC cell migration. A detailed analysis of extracellular matrix composition and organization over time as well as a clear quantification of the roughness index of the NC migratory environment would need to be performed with modern tools. Even if repeated topological biases at microscopic scale would be observed it is unclear how such biases would be implemented and maintained in 4D throughout head morphogenesis to sustain directed NC migration over time. In addition to MMP14 discussed above, MMP2, 3, 7, 9, 11, 13, 15, 16, 18, 20, 24, and 28, as well as multiple ADAMs, are expressed by cephalic NC cells or produced by the environment they cross during migration (see Christian et al., 2013, Table 1 in Gouignard et al., 2020 and references therein). Thus, in this context, the likelihood of relatively stable and iteratively distributed topological or mechanical cue (a requirement for ratchetaxis) along the dorsoventral path of cephalic NC migration appears quite low.

## Galvano/Electrotaxis

Another mechanism that can generate directed cell motion is the detection of electric fields, known as galvanotaxis (or electrotaxis). Interestingly, in mammalian cell lines, lipid rafts were shown to take part in galvanotaxis (Lin et al., 2017) and electric fields also affect the GSK3β-dependent polarization of the Golgi apparatus (Cao et al., 2011) which helps organizing the non-centrosomal microtubule network, a key player in front–rear cell polarity (Meiring et al., 2020). GSK3β is required for cephalic NC migration in Xenopus (Gonzalez Malagon et al., 2018) and is a known regulator of Snail cytoplasmic-nuclear shuttle (Muqbil et al., 2014). Thus, by regulating C3aR/CXCR4 carrying lipid rafts and GSK3β, electric fields might be acting on multiple levels during Xenopus NC cell migration: EMT, front-rear polarity, cell–cell, and cell-matrix adhesions. The ability of trunk NC cells to undergo galvanotaxis was shown using quail, Xenopus and axolotl embryos trunk neural tube explants *in vitro*, which were sometimes cultured for days before fields were applied (Stump and Robinson, 1983; Cooper and Keller, 1984; Nuccitelli and Smart, 1989; Gruler and Nuccitelli, 1991; Nuccitelli et al., 1993). However, to our knowledge, electrotaxis has not been assessed in primary cephalic Xenopus NC cell culture. Some of the behaviors described in the literature appear to be somewhat artefactual with cells permanently elongated perpendicularly to the applied field. One of the reason may be the strengths of the applied electric fields used ranging from 100 to 600 mV/mm (Nuccitelli and Erickson, 1983; Cooper and Keller, 1984) which are 4–22 times higher than what has been measured *in vivo* in Xenopus (Hotary and Robinson, 1994). Indeed, from early in development, the Xenopus embryo has a transepithelial potential and electrical currents (Hotary and Robinson, 1994). An anteroposterior gradient is detected from the blastopore and applying electric fields to nullify it led to developmental defects such as failure of anterior neural tube closure and reduced head development. Noticeably, it led to expulsion of cells from the blastopore which might indicate that the anteriorward displacement of mesoderm is partially affected. Given that this movement is crucial to generate a stiff environment for cephalic NC cells to migrate (Barriga et al., 2018), one could propose that the observed head defects in embryos with nullified electric fields might be due to a partial failure of cephalic NC migration linked to improper mesoderm development. As for the other putative guiding mechanisms discussed, electric fields will not be a one-size-fit-all cue. While most cell types exposed to electric fields seem to migrate toward the cathode, some, such as macrophages, seem to prefer the anode (Sun et al., 2019). Also, as discussed for the other taxis, some of the cellular structures required for sensing and implementation of a polarity bias at the single cell level are not specific to electric fields as an input (e.g., lipid rafts, cell surface receptors).

## Conclusion

All these interplays are mind blowing and place us, as experimentalists, in a chicken and egg situation. Hierarchy between signals and pathways is difficult to dissect because of the numerous cross-regulations taking place during migration itself. Exposure to chemokines is needed for cell-matrix adhesion. Cell-matrix adhesions are needed for motility and mechanosensing. Mechanosensing controls nuclear shuttling of transcription factors. These factors control expression of adhesion molecules and cytoskeleton components which in turn feedback into cell polarity, etc. Therefore, rather than being driven by competing guidance strategies, cephalic NC cells seem to iteratively use the molecular machinery of cell motility and adhesion to read the various signals at their disposal. This blurs the lines between the different kinds of taxis even if for most of them the initial cue is clearly identifiable (e.g., chemokine, rigidity, electric field). This may mean that an understanding of the complexity of an *in vivo* morphogenetic process such as NC cell migration requires a systems biology approach with contribution from multiple disciplines to integrate studies in which cues, genes or pathways are handled one at a time. We can think of it as studying the role that each individual LEGO piece plays in forming a bigger structure. Taking a single piece out is extremely powerful to gather information about it. However, at some point, one needs to try to fit all pieces together. The added difficulty is that in the regulation of *in vivo* cell migration each LEGO piece has melted and started to blend with several of its direct neighbors.

Our aim with this review is to raise awareness about artificial distinctions between supposedly different modes of cell guidance. In that context, we (as a community of NC researchers) should always keep in mind that the signal we are looking at in a given project may actually influence other inputs. The reason for that is that NC cells are exposed to multiple signals and may have evolved to use them all at once, not one by one. That is already a fact based on published data but we probably underestimate it. Thus, we might need to systematically assess what knocking down one input does “outside” of its expected canonical function and with that in mind, design appropriate controls for our experimental approaches. We believe that the point we are making here invites the field to leave the current comfort zone and to address directed cell migration both in the context where it takes place and with the complexity it deserves.

## Author Contributions

EB and ET designed and wrote the review. Both authors contributed to the article and approved the submitted version.

## Conflict of Interest

The authors declare that the research was conducted in the absence of any commercial or financial relationships that could be construed as a potential conflict of interest.
